# Fluorination: A strategy to tune the interactions of molecules and nanoscale systems with biological matter

**DOI:** 10.1002/2211-5463.70354

**Published:** 2026-10-08

**Authors:** Martina Beccalli, Beatrice Lucia Bona, Francesca Baldelli Bombelli

**Affiliations:** ^1^ Laboratory of Supramolecular and Bio‐Nanomaterials, Department of Chemistry, Materials, and Chemical Engineering “Giulio Natta” Politecnico di Milano Milan Italy; ^2^ Department of Medical Biotechnology and Translational Medicine University of Milan Segrate Italy

**Keywords:** bio–nano interaction, drug delivery, fluorination, self‐assembly

## Abstract

Fluorination has become a key molecular engineering strategy in medicinal chemistry and nanomedicine, enabling the fine tuning of the physicochemical and biological properties of therapeutic molecules and drug delivery systems. The unique characteristics of fluorine, including its small steric footprint, high electronegativity, and exceptional strength of the C–F bond, enhance metabolic stability, modulate lipophilicity and acidity, and improve pharmacokinetic profiles. Beyond these well‐established effects, fluorination has emerged as a tool for controlling the assembly and biological behavior of nanocarriers. Fluorinated biomolecules, polymers, dendrimers, and lipid‐based systems often display improved colloidal stability, reduced nonspecific protein adsorption, enhanced cellular uptake, and efficient endosomal escape, ultimately leading to superior intracellular delivery of nucleic acids, proteins, and other therapeutic cargoes. Here we summarize recent advances in the fluorination of both therapeutic cargoes and delivery carriers, emphasizing how different fluorination strategies, including single fluorine atoms, trifluoromethyl groups, linear perfluoroalkyl chains, and emerging short branched fluorinated motifs, can be exploited to optimize drug delivery performance. Attention is devoted to the relationship between fluorinated molecular architectures, self‐assembly, and bio–nano interactions that govern biological activity. Furthermore, the advantages and limitations of current fluorination approaches are discussed. Finally, we highlight the need for a deeper mechanistic understanding of how fluorinated moieties interact with proteins and biological membranes. Advanced physicochemical techniques, including neutron and X‐ray scattering and reflectometry, are expected to provide fundamental insights into these processes, enabling the rational design of more effective, safer, and environmentally sustainable fluorinated therapeutics and nanomedicines.

## Graphical abstract

This minireview explores fluorination as a key strategy to optimize therapeutic molecules and nanocarriers. By incorporating different fluorinated motifs—from single atoms to linear and branched chains—drug delivery, cellular uptake, and colloidal stability can be improved and fine‐tuned. We highlight recent advances, focusing on the physicochemical effect of fluorination on stability and bio–nano interactions.

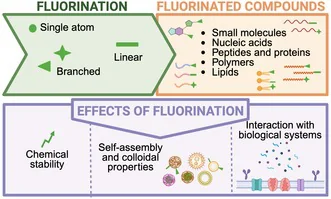

**Abbreviations**
CPP: Cell penetrating peptides
DSPE: 1,2‐distearoyl‐[sn]‐glycero‐3‐phosphoethanolamine
EE: Encapsulation efficiency
FANA: 2′‐deoxy‐2′‐fluoroarabinonucleic acid
FBS: Fetal bovine serum
FDA: Food and Drug Administration
HFBA: Heptafluorobutyric acid
LNP: Lipid nanoparticle
MRI: Magnetic resonance imaging
NA: Nucleic acid
NMR: Nuclear magnetic resonance
NP: Nanoparticle
PAGE: Polyacrylamide gel electrophoresis
PAMAM: Poly(amidoamine)
PCL: Poly ε‐caprolactone
PEG: Polyethylene glycol
PEI: Polyethylenimine
PFtB: Perfluoro‐*tert*‐butyl
PLGA: Poly(lactic‐co‐glycolic acid)
PPI: Poly(propylene imine)
PTFE: Polytetrafluoroethylene
SNA: Spherical nucleic acid

## Introduction

Fluorination is a promising strategy to tune the properties of organic compounds and improve their biological activity. Fluoro‐organic molecules are nowadays highly occurrent in medications. First, the fluorine atom (van der Waals radius 1.47 Å) is just slightly larger than the hydrogen atom (van der Waals radius 1.20 Å), so the molecular structure is not significantly impacted when H is replaced by F. Second, the C‐F bond is the strongest link made by carbon, so fluorine substitution on metabolically labile sites can be used to hinder hydrolysis and enzymatic oxidation processes, therefore improving metabolic stability [1, 2]. Third, given its electronegativity, F induces bond polarization, hence affecting the lipophilicity/hydrophilicity of a compound [1]. For these reasons, fluorination has been widely used in medicinal chemistry, with 20% of current drugs on the market containing fluorine. Adding a fluorine atom or trifluoromethyl group (‐CF_3_) in a drug molecular structure enhances its lipophilicity, chemical stability, binding affinity, and pharmacokinetics, often resulting in better therapeutic profiles [3]. Fluorination can be exploited to modulate the acidity and basicity of molecules, leading to fine tuning of binding affinity, pharmacokinetics, and bioavailability. Substitution of hydrogen with fluorine on aromatic rings slows down considerably the oxidative metabolic step of a molecule, hence affecting its metabolic stability [1, 2, 3]. Fluorinated compounds are known for their high hydrophobicity and lipophobicity, a phenomenon known as the ‘*fluorous effect*’, that is, attitude of fluorinated chains to preferentially give *fluorine–fluorine* interactions and segregate [4]. Beyond its established role in enhancing drug stability and pharmacokinetics, fluorination has also emerged as a powerful strategy for modulating bio–nano interactions in nanoscale assemblies, thereby promoting more efficient cytosolic delivery of nucleic acids (NA) and proteins [5, 6]. Analogously, self‐assemblies formed by fluorinated amphiphiles showed higher colloidal stability, both in physiological conditions and in biological environment [7]. It has been shown that fluorination of gene delivery platforms strongly improved their cellular uptake and gene endosomal escape: by 3‐ to 10‐fold for polymer [8] and by 30‐ to 50‐fold for lipid‐based carriers [6, 9]. While the benefits of fluorination on NA delivery are evident, the underlying mechanisms are not fully understood. In particular, the capabilities of fluorinated delivery vehicles compared to their nonfluorinated counterparts are attributed to the limited interaction of fluorinated vehicles with plasma proteins and to the lipophobic nature of fluorinated groups, which hinder fusion with lipid membranes creating segregated fluorinated domains and destabilizing them [10, 11]. Previous fluorination strategies primarily focused on incorporating ‐CF_3_ groups or long linear perfluoroalkyl chains, with the latter raising concerns regarding accumulation and clearance times [12]. More recently, short, branched fluorinated chains—considered more biocompatible and less prone to tissue accumulation—have been exploited, while also providing magnetically equivalent ^19^F atoms for imaging applications such as ^19^F‐MRI and Raman microscopy [13, 14].

In this minireview, we highlight recent advances in the fluorination of biomolecules and self‐assembling amphiphilic systems—including polymers, dendrimers, and lipids—and discuss how these modifications can be leveraged to finely tune their physicochemical properties and interactions with biological environments. Emphasis is placed on understanding fluorination from the dual perspective of both cargo and carrier within drug delivery platforms. By examining how fluorinated motifs influence assembly behavior, stability, cellular uptake, and intracellular trafficking, we aim to provide a comprehensive overview of how fluorination can be strategically employed to enhance the performance of next‐generation delivery systems.

### Fluorination of drug delivery platforms: Cargoes and carriers

Efficient intracellular internalization and cytosolic delivery are essential for therapeutic molecules and nanosystems to exert their biological activity, as many therapeutic targets are located within the cell. Consequently, enhancing cellular uptake and overcoming intracellular barriers are key challenges in the development of advanced delivery platforms. These aspects can be addressed by modifying either the therapeutic agents (cargoes) or their delivery systems (carriers), improving the physico‐chemical properties of the former and colloidal stability and delivery performance of the latter [3, 15].

Multiple fluorination strategies are available to tune the properties of molecules and nanostructures, as shown in Fig. 1. Single fluorine atoms and –CF_3_ groups are widely used to modify cargo molecules, affecting their binding affinity and thus improving their stability in biological media, leading to reduced degradation and enhanced cytosolic delivery [3, 16]. Functionalization with fluoroalkane chains and branched fluorinated moieties are largely used strategies for both cargoes and carriers, and fine tuning of the properties can be obtained by choosing the degree of fluorination [17, 18]. Fluorination can induce two different main effects depending on the distribution of the fluorinated groups (Fig. 1). Fluorinated compounds tend to segregate due to the ‘*fluorous effect*’, causing self‐assembly into nanostructures with a fluorinated core that exhibit enhanced colloidal stability in biological fluids [17, 18, 19]. However, when fluorinated moieties are exposed to the surrounding environment, they play a fundamental role in the interaction with biological fluids and cellular membranes [3, 8]. For example, fluorination of the NP surface affects the formation of the protein corona, improving the resistance to protein binding [8]. Of note, the interaction of fluorinated platforms with proteins should be investigated case by case since it may also depend on other factors. Moreover, fluorination also affects the interactions with cellular membranes. Fluorinated compounds have a strong tendency to adsorb on cell membranes due to low fluorine‐water interactions, and favorable cell adhesion is beneficial for efficient cellular uptake [17, 20, 21]. Additionally, the limited miscibility between fluorinated compounds and phospholipids can minimize the fusion with the cell membrane during endocytosis, avoiding not only long‐term retention on the cell membrane, but also membrane disruption and cytotoxicity [20]. Limited fusion of fluorinated molecules with the cell membrane leads to higher membrane permeability, therefore to larger cellular internalization and endosomal escape [17].

**Fig. 1:**
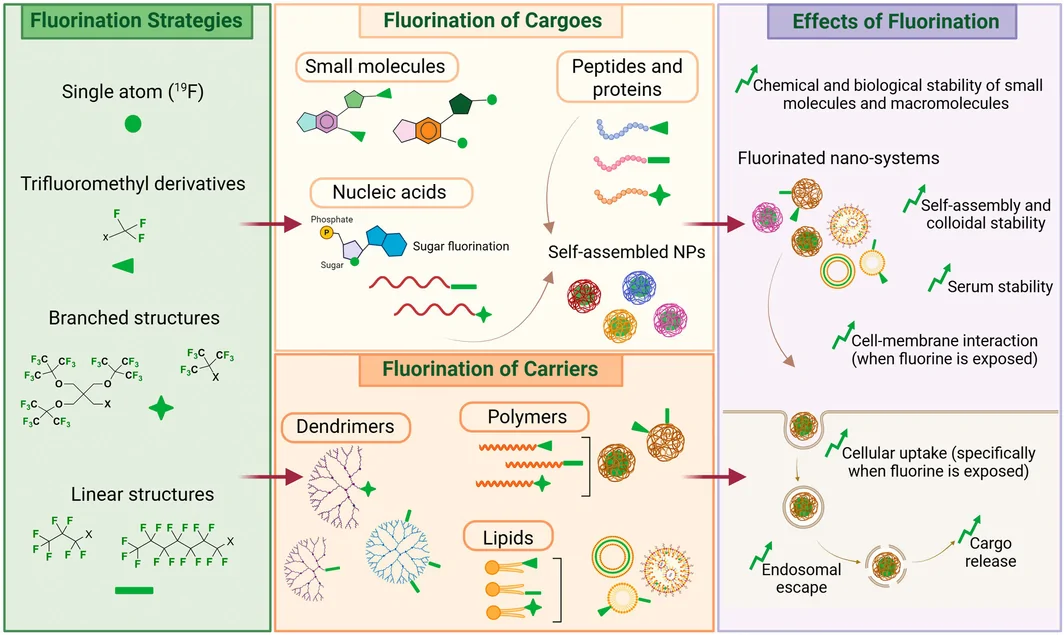
Schematic illustration of fluorination methods, applications for both cargoes and carriers, and fluorination effects. Created in BioRender. Bona, B. (2026) https://BioRender.com/c038qf9

### Fluorination of cargoes

#### Small molecules

Fluorine has been widely used in the medicinal field since 1954, when the corticosteroid *florinef* acetate was introduced on the market [3], followed by the clinical implementation of 5‐fluorouracil for cancer treatment in 1957 (Fig. 2A) [2, 22]. In the 1980s, fluoroquinolones were established as antibacterial agents. Given these successes, the number of fluoropharmaceuticals receiving FDA approval has spiked over the last two decades, and nowadays, approximately one‐third of the best performing drugs contain fluorine atoms [3]. The success of the introduction of fluorine atoms in drug molecules is related to the peculiar properties of the C‐F bond, such as high bond strength, polarity, and minimal steric hindrance of F atoms. In particular, the introduction of fluorine atoms, characterized by high electronegativity, alters the electron distribution in the molecule [23], inducing a decrease in the pKa [1, 24, 25], an enhanced bioavailability [1, 23] and a reduced cytotoxicity [23]. For example, the addition of fluorine to methotrexate increased the acidity of the neighboring carboxyl group, reducing polyglutamylation and thus effectively decreasing cytotoxicity [23]. Moreover, on 3‐piperidinylindole antipsychotic drugs, fluorination decreased the basicity of the amines, therefore showing greater affinity to the 5‐HT_2A_ receptors over the dopamine D_2_ receptor and leading to a huge improvement in bioavailability and binding affinity [1].

**Fig. 2:**
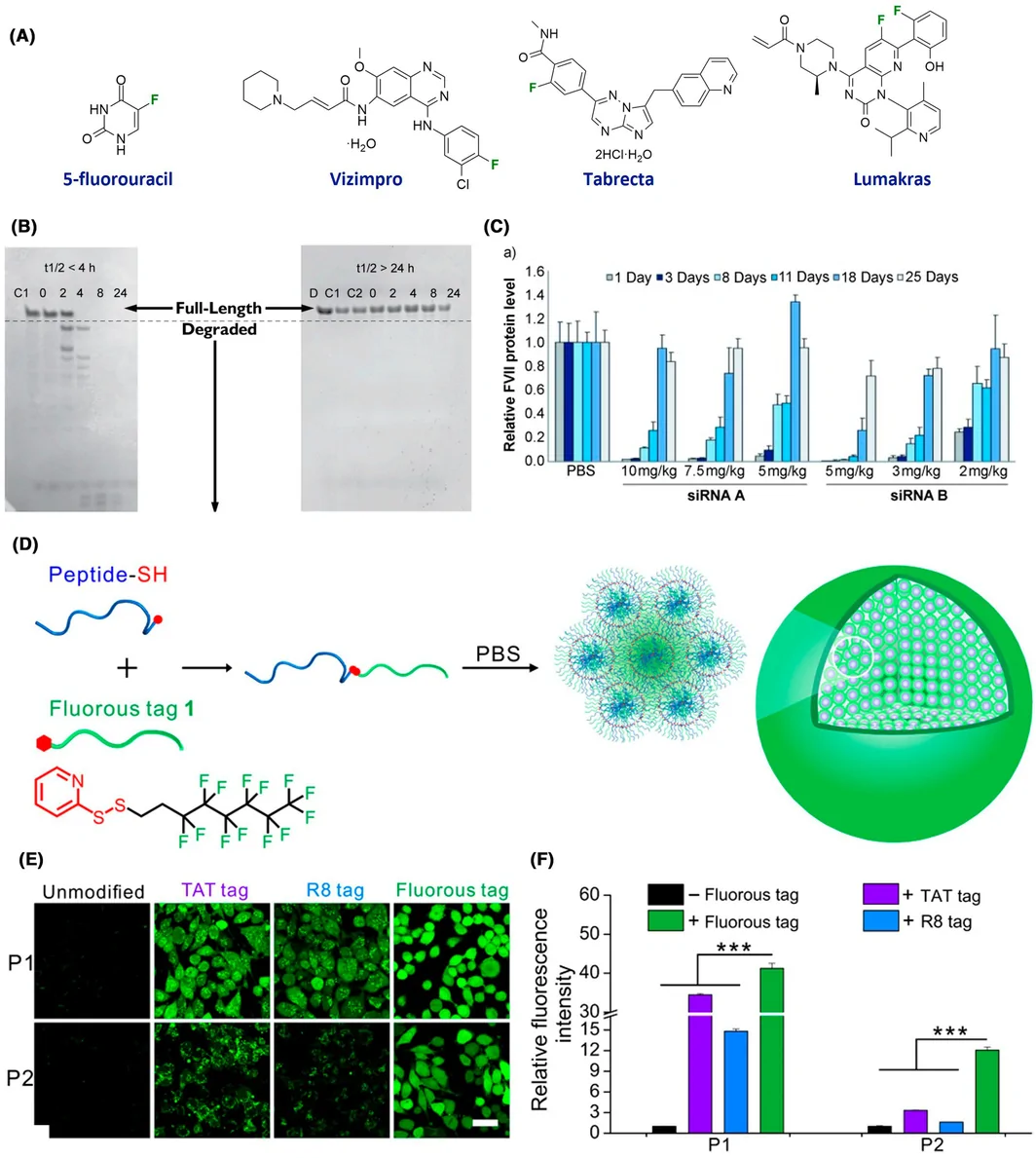
Fluorination strategies of cargo molecules. (A) Molecular structure of fluorinated drugs. (B) Polyacrylamide gel electrophoresis (PAGE) images of nonfluorinated (left) and fluorinated (right) siRNA incubated in human serum at 37 °C for different times. Whereas fluorinated siRNA exhibits only the band for the intact molecule up to 24 h, nonfluorinated siRNA shows bands indicating complete nucleic acid (NA) degradation within 4 h. (C) Therapeutic effect of fluorinated siRNA (B) and control siRNA (A) iv administered as lipid nanoparticles (LNP) to mice (*n* = 5). FVII protein levels were measured from serum collected at the indicated time points postadministration by using a chromogenic assay (Coaset Factor VII, DiaPharma Group or Biophen FVII, Aniara Corp.) (D) Scheme of peptide fluoroalkylation and induced self‐assembly into nanostructures; (E) Confocal images of HeLa cells treated with peptides after 6 h (scale bar, 40 μm). Unmodified, TAT‐modified, and R8‐modified peptides were tested as controls. Peptides (10 μm) were tested for each sample. (F) Relative fluorescence intensity of cells treated with peptides in (E). Adapted from [32] (B,C) and [17] (D, E, F).

The unique properties of fluorinated compounds have also been merged with heterocyclic frameworks, which are present in many natural products and bioactive compounds, yielding a new class of biologically relevant molecules with enhanced metabolic stability and tunable properties [3, 26]. Representative examples include *enoxacin*, for urinary tract infections and *favipiravir* (approved in 2014 in Japan) as an antiviral drug [26]. In oncology, the integration of fluorine atoms and trifluoromethyl (‐CF_3_) groups onto aromatic rings, as seen in drugs for nonsmall‐cell lung cancer treatment, such as *Vizimpro*, *Tabrecta*, and *Lumakras*, whose structure is shown in Fig. 2A, improves metabolic stability, membrane permeability and binding affinity to the target protein via nonbonding ligand–protein interactions involving fluorine [27]. Indeed, the C‐F bond is highly polarized, and thus, it should interact with other molecules through dipole–dipole and dipole‐charge interactions. Furthermore, if incorporated correctly, fluorine atoms can be used to tune the molecular conformation, increasing the drug affinity to its protein target [28]. Similarly, the introduction of a geminal difluoro group on cyclic or aliphatic systems (e.g., *Tibsovo* and *Scemblix* for myeloid leukemia) enhances drug potency and metabolic resistance [27]. Furthermore, fluorinated drugs such as *Cabenuva*, *TPOXX*, and *Xofluza* have been recently approved by FDA to treat HIV, smallpox, and influenza, respectively [27].

#### Biological therapeutic agents

In addition to drugs, therapeutic agents include biologics such as NAs and peptides. NAs have gained a large interest due to their ability to silence disease‐related genes efficiently and selectively [29, 30], whereas peptides can target specific protein sites, acting against infections and cancer [5, 17]. However, both NAs and peptides suffer from degradation in biological media and poor cellular internalization [5, 17, 29, 30]. Fluorination has emerged as a strategy to enhance the cytosolic delivery of these biologics by mediating interactions with serum proteins, improving cell membrane adhesion, and reducing fusion with cellular membranes [20]. Depending on the specific fluorination approach, improved metabolic stability and cellular internalization were observed for these macromolecules as a consequence of enhanced chemical stability and/or self‐assembly induced by the presence of fluorinated moieties [16, 17, 18, 31]. For example, the introduction of a single fluorine atom in NAs resulted in modification of binding affinity and improved chemical stability [16, 32], while the addition of F atoms or ‐CF_3_ groups to peptides promoted a secondary structure [31, 33, 34, 35]. On the other hand, functionalization with larger fluorinated groups renders these macromolecules amphiphilic, promoting their self‐assembly, a process that can be amplified by the ‘*fluorous effect*’, as observed for both NAs and peptides [17, 18, 36].

Regarding NAs, chemical modification is a crucial strategy to improve their therapeutic efficacy. In particular, 2′‐deoxy‐2′‐fluoro (2′‐F) modification has been widely used to modify NAs [32], and it was recently exploited to improve their serum stability and transfection efficiency [16, 32]. In a work by Manoharan et al., 2′‐F‐modified siRNA was found to have a serum half‐life greater than 24 h, whereas the nonfluorinated siRNA was completely degraded within 4 h, as demonstrated by polyacrylamide gel electrophoresis (PAGE) of siRNA incubated in human serum at 37 °C at different times (Fig. 2B). Moreover, 2′‐F‐modified siRNA was roughly twice as potent as the unmodified control in terms of transfection both *in vitro* and *in vivo*. The relative FVII protein level expressed by mice at different time points is shown in Fig. 2C, indicating that, when injected with the same dose of siRNA‐loaded LNP, 2′‐F‐modified siRNA led to halved FVII protein levels compared to nonfluorinated siRNA [32]. siRNAs have also been fully modified with an alternating 2′‐fluoro/2′‐*O*‐methyl pattern, inducing the formation of an A‐form helical structure [33]. The impact of this full modification was significantly less pronounced *in vitro* than *in vivo*, where fully modified siRNAs are retained at 100x higher levels in various tissues, independently of the sequence, promoting mRNA silencing [37]. 2′‐F modification was also exploited to create 2′‐deoxy‐2′‐fluoroarabinonucleic acid (FANA), a promising DNA‐like nucleic acid adaptable for numerous biological applications [16].

Considering the ability of FANA/RNA hybrids to elicit RNase H activity, RNA cleavage rates and efficiency were investigated and a PS‐FANA‐DNA 1–1 altimer was found to be more potent than the corresponding gapmer. FANAs were also mixed with 2′‐modified RNA, resulting in potency comparable to the one of unmodified RNA, but with the benefits of higher nuclease resistance and reduced immune response [16]. However, fluorination is also a powerful strategy to modify spherical nucleic acids (SNAs) ‐ supramolecular nanostructures with a core (liposomal, metallic or polymeric) surrounded by a dense NA shell [18]. Linear [36, 38] and branched [18] fluorinated moieties have been used to optimize SNAs, resulting in both cases in facilitated self‐assembly due to the fluorine–fluorine interactions in the core of the SNA. Fluorinated SNAs exhibited enhanced cellular uptake [36] and unmatched levels of gene silencing *in vitro* compared to nonfluorinated SNAs [18]. The degree of fluorination was shown to be responsible for self‐assembly properties and kinetics of the NA release, hence providing a tunable platform for cytosolic delivery applications [18, 36].

In the realm of therapeutic peptides, instead, traditional methods to facilitate cytosolic delivery, such as conjugation to cell‐penetrating peptides (CPPs) or complexation with polymers, fail to address both intracellular and extracellular obstacles simultaneously [17]. Fluorination has thus emerged as a potent method to improve their thermal and chemical stability, triggering the formation of stable secondary structures in aqueous solutions that exhibit higher affinity to lipid membranes [31, 33, 39]. Multiple works focused on the addition of F atoms and fluorinated groups on specific amino acids, showing that fluorination induces formation of secondary structures, promoting the formation of β‐sheets thanks to the decreased polarity and enhanced hydrophobicity [31, 35]. In addition, fluorinated peptides exhibit ^19^F‐MRI signal, hence being suitable tools for imaging [40]. Considering peptide hydrogels for drug delivery applications, it was proved that adding halogen atoms can influence the π–π interactions, leading to mechanically stronger gels [41]. Systematic substitution of F atoms and ‐CF_3_ groups on a heptapeptide was investigated. An increase in fluorine content could be partially related to an overall increase in hydrophobicity, and introducing fluorine atoms was shown to improve hydrogel mechanical properties, resulting in more rigid hydrogels [35, 41]. However, fluorination must be tuned to achieve better properties [41]. As a general rule, incorporation of fluorinated groups leads to higher storage modulus *G*′ [41]. However, both number of F atoms and their position on the aromatic ring were shown to significantly impact the mechanical properties of fluorinated peptides, and the ones bearing only one phenylalanine with a fluorinated group exhibited excellent G" values, possibly due to improved stacking between a fluorinated ring and its neighboring nonfluorinated one. *In vivo* gel stability experiments showed slower gel erosion for the fluorinated hydrogels compared to the nonfluorinated counterpart, therefore indicating fluorinated peptide hydrogels as promising candidates in the field of controlled release [41]. Trifluoromethyl groups were also used to functionalize antimicrobial peptides, showing once again how design and tuning of the functionalization can effectively improve antimicrobial properties due to formation of stable secondary structures [33, 34]. Fluoroalkyl chains, in particular ‐(CF_2_)_5_CF_3_, have also been used as fluorous tags on cargo peptides (Fig. 2D). Due to the fluorine–fluorine interactions, fluorinated peptides self‐assembled into more uniform nanostructures compared to the nonfluorinated counterparts. Moreover, the assembly of fluorinated peptides into highly stable nanostructures protected them against protease degradation. *In vitro* tests showed that fluoroalkylation promoted peptide cytosolic delivery, displaying superior efficiency in comparison to CPPs. As shown by confocal images in Fig. 2E and by the graph in Fig. 2F, peptides with the fluorous tag exhibited higher fluorescence intensity compared to unmodified, TAT‐modified and R8‐modified peptides at the same concentration. In addition, confocal images (Fig. 2E) show that TAT‐ and R8‐modified peptides exhibit punctuate distribution in cells, indicating entrapment by endosomes and lysosomes. On the other hand, fluoroalkylated peptides show distributed fluorescence throughout the cytosol, suggesting successful endosomal escape [17].

#### Fluorination of carriers

A second effective strategy for optimizing cytosolic delivery involves encasing the unmodified cargo inside a protective vector to shield the therapeutics from the enzymatic degradation in biological fluids. However, Perfluoro‐tert‐butyl groups are being explored as *relative* alternatives to longer‐chain fluorinated groups because their shorter, branched architecture can reduce some of the bioaccumulation and persistence concerns associated with conventional long‐chain perfluoroalkyl groups, while retaining useful, fluorinated properties. while the cargo is protected from degradation, carriers still face several challenges, such as short blood circulation times, serum instability, rapid clearing [15], and poor targeting capabilities [13].

Fluorination of delivery carriers for drugs and biologics has been widely recognized as an effective strategy for improving their performance. This enhancement is again attributed to the superior colloidal stability of self‐assembled fluorinated nanostructures and, in certain cases, to their greater efficiency in encapsulating hydrophobic drug molecules [42]. While conventional nanocarriers often suffer from drug leakage and premature release, introduction of fluorine atoms can withdraw electron density from reactive groups, hence reducing hydrolysis rate and extending circulation time [1, 2]. Conversely, when fluorine is exposed on the surface of the carrier, its omniphobic nature reduces nonspecific protein adsorption, thereby avoiding the formation of a stable protein corona and recognition by the immune system, leading to longer circulation times [41]. Furthermore, fluorinated nanostructures preferentially adsorb onto cellular membranes to reduce fluorine‐water interfacial interactions, enhancing their cellular internalization. However, their lipophobic character limits fusion with the cell membrane during endocytosis, in contrast to the typical behavior of hydrogenated carriers [13]. Another notable advantage of fluorinated systems is their ability to serve as imaging agents [43, 44, 45]. Additionally, when fluorine atoms are magnetically equivalent, they produce a strong ^19^F‐MRI signal, thereby conferring excellent bioimaging functionality to the nanocarriers [15, 44].

Fluorination has been extensively applied to soft nanocarriers such as polymers, dendrimers, and lipids, typically using short linear perfluoroalkyl chains (from CF_2_‐CF_3_ to (CF_2_)_6_‐CF_3_) to enhance self‐assembly, serum stability, and interactions with cellular membranes while minimizing toxicity [5, 46, 47, 48, 49, 50, 51]. The positioning of fluorinated moieties depends on the carrier type: They are generally located at the termini of dendrimer branches [5, 8, 52] and as side chains in polymers, exposing fluorinated groups on the nanoparticle surface and reducing protein corona formation through fluorine's omniphobic character [6, 10, 47, 48, 53, 54] In contrast, fluorinated segments in lipid‐based carriers are incorporated within the lipid heads or tails, where they primarily improve self‐assembly and cargo encapsulation rather than surface properties [5, 8, 47, 53].

Despite their functional advantages, linear perfluoroalkyl chains longer than six ‐CF_2_‐ units raise concerns due to environmental persistence, bioaccumulation, and toxicity [53, 55, 56, 57, 58]. Perfluoro‐tert‐butyl groups are being explored as *relative* alternatives to longer‐chain fluorinated groups because their shorter, branched architecture can reduce some of the bioaccumulation and persistence concerns associated with conventional long‐chain perfluoroalkyl groups, while retaining useful fluorinated properties [58, 59]. Their high density of terminal CF_3_ groups lowers surface energy, enhances hydrophobicity, and provides superior ^19^F‐MRI performance by generating stronger, less split fluorine signals due to the presence of magnetically equivalent ^19^F atoms. Consequently, branched fluorinated moieties have been increasingly incorporated into polymers, dendrimers, and lipid‐based nanocarriers to improve serum stability, transfection efficiency, and imaging capabilities.

#### Polymers

Polymer‐based nanoparticles have been widely investigated for small molecules, gene, and protein delivery [60]. Fluorinated polymers have been largely investigated since polytetrafluoroethylene (PTFE) was discovered in 1938. Owing to the combination of the carbon–carbon and carbon–fluorine bonds, these polymers show high thermal stability and chemical resistance, which can be tailored by adjusting the fluorine content [61].

The interesting properties of fluoropolymers, combined with their simplicity in formulation, led to applications in the biomedical field in delivery systems [58]. The addition of fluoroalkane chains to polymer‐based NA carriers led to exceptional self‐assembly behavior and stability in biological fluids [61, 62, 63]. Functionalization of a PEG‐11 block with (CF_2_)_7_CF_3_ led to spontaneous self‐assembly into stable nanoparticles in aqueous solutions. The fluorinated nanoparticles were tested both *in vitro* and *in vivo*, revealing low cytotoxicity and lack of long‐term accumulation, suggesting suitability for biomedical applications [62]. PEG functionalized with longer perfluoroalkyl chains, namely (CF_2_)_12_CF_3_, was shown to self‐assemble into small micelles (~12 nm), while also exhibiting excellent oxygen transport capabilities thanks to the perfluorinated core, suggesting the possibility of being used as oxygen shuttles to overcome tumor hypoxia and thus improve ionizing radiation efficiency [63]. With this application in mind, perfluorinated micelles were found to be not cytotoxic in absence of radiation, while the effect of γ rays was significantly increased by the presence of micelles [63]. Moreover, these nanosystems were radiolabeled [63, 64] or further optimized by encapsulating PERFECTA to be ^19^F‐MRI sensitive, thus allowing for noninvasive imaging [65, 66].

Multiple studies showed how introducing fluorinated side chains in polymers, such as chitosan and PEI, led to enhanced cytosolic delivery compared to their nonfluorinated counterparts, possibly due to reduced formation of protein corona thanks to fluorine exposure on the surface [42, 44, 59]. Conversely, when the polymer was pegylated and functionalized with fluorinated side chains, PEG was exposed on the surface, while side fluoroalkyl chains stabilized the core of the carrier due to fluorous interactions, as shown schematically in Fig. 3A. The obtained NPs showed enhanced serum stability and excellent efficacy even in the presence of serum. The fluorinated nanocarriers were shown to facilitate NP cellular uptake, intracellular distribution, and NA endosomal escape compared to the nonfluorinated benchmark, achieving excellent silencing activity even in the presence of serum proteins up to 30% FBS, as shown by the values of Luc expression (%) in Fig. 3B. In contrast, control PEI polyplexes lost their silencing activity with increasing serum content, resulting in only 3% silencing activity at 30% FBS in B16F10 cells [10]. However, while longer fluoroalkyl chains usually lead to higher delivery efficacy, an excessively long chain can result in extreme fluorophilicity inducing pre‐assembly of fluoroamphiphiles and reduce the cargo encapsulation efficiency. Therefore, fluoroalkyl chain length must be optimized to get a beneficial fluorophilicity balance for efficient delivery [58].

**Fig. 3:**
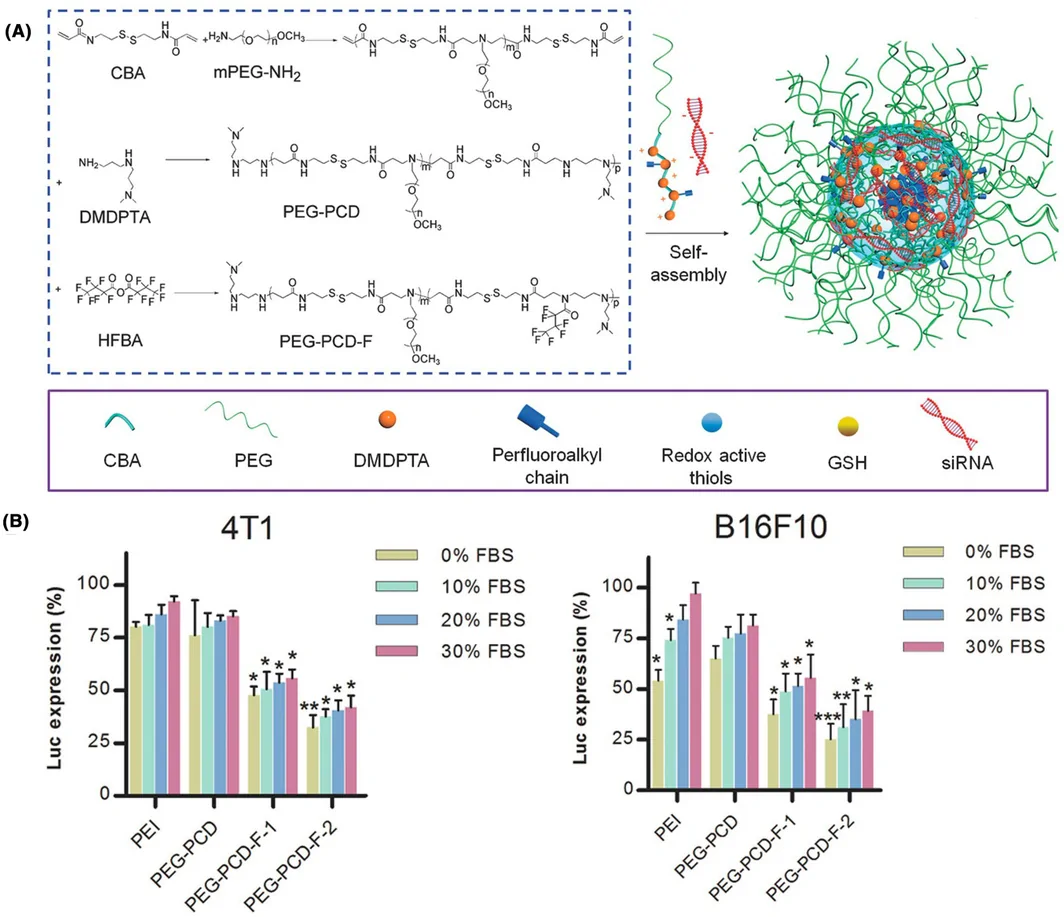
Structure of fluorinated polymers and gene silencing activity. (A) Synthesis of PEGylated (poly(ethylene glycol)) fluorinated polymers and scheme of their self‐assembly with siRNA to form polyplexes; (B) Effect of fetal bovine serum (FBS) on reporter Luc gene silencing ability of polyplexes on different cell lines (4 T1 and B16F10): fluorinated dendriplexes PEG‐PCD‐F‐1 (5 wt% F) and PEG‐PCD‐F‐2 (9 wt% F) are compared to PEI and PEG‐PCD as controls. *P* < 0.05, **P < 0.01, ***P < 0.005 versus untreated group (set as 100%). Results shown as mean ± SD (*n* = 3). Adapted from [10].

In this context, shorter, branched fluorinated moieties have been explored as alternatives to long, linear perfluoroalkyl chains, enabling the development of biocompatible polymer‐based carriers that retain comparable properties while also providing ^19^F‐MRI capability. Various polymers containing ‐(CF_3_)_3_ (PFtB) moieties have been synthesized, and their self‐assembly properties and transfection efficacy have been investigated [5, 55, 60, 61]. For hydrophilic polymers such as PEG, high degrees of fluorination are necessary to induce self‐assembly into nanostructures since ‘fluorous’ interactions are stabilizing the whole structure [67]. The effect of PFtB was also studied for hydrophobic polymers such as poly(lactic‐co‐glycolic acid) (PLGA) and poly ε‐caprolactone (PCL). PFtB‐PLGA NPs showed improved encapsulation efficiency (EE%) of hydrophobic drugs containing ^19^F‐atoms compared to the nonfunctionalized control. These results did not arise from direct fluorine–fluorine interactions between cargo and carrier, but rather from the increased affinity of hydrophobic drugs for the fluorophilic environment of the system, regardless of whether the drugs themselves were fluorinated [42]. Instead, fluorine–fluorine interactions among PFtB‐modified PEGylated PCL NPs were responsible for their higher colloidal stability in the biological environment [7]. Further *in vitro* and *in vivo* studies are needed to determine the real efficacy of these systems, but the higher EE% and enhanced serum stability suggest a possible improvement in therapeutic efficiency. Moreover, PFtB‐functionalized nanocarriers exhibited intense ^19^F‐NMR signals, therefore being promising theranostic drug delivery vectors [7, 42].

#### Dendrimers

Since they were conceived in the late 1970s and early 1980s, dendrimers have been widely investigated for both drug and gene delivery applications [68, 69]. Their large use as delivery agents is due to their unique properties such as a highly branched 3D architecture, well‐defined size and high density of terminal functional groups [68, 69]. However, as gene delivery carriers they can suffer from low transfection efficiency and cytotoxicity (linked to their usual high positive charge density) [54, 70]. Introduction of fluoroalkanes on PAMAM and PPI cationic dendrimers has been proved to be an effective strategy to reduce cytotoxicity and improve cytosolic delivery [8, 54, 70, 71]. Multiple works investigating different generations of HFBA‐modified PAMAM and PPI dendrimers demonstrated that the highest transfection efficacy is obtained with a tailored balance of the degree of fluorination, by tailoring both length and number of fluoroalkyl chains [8, 54, 71]. These beneficial effects are attributed to the distinct bio–nano interactions of fluorinated systems relative to their unmodified counterparts, although the underlying mechanisms have yet to be fully elucidated [5, 8, 54]. One of the most significant results in this field is obtained by an HFBA‐modified G5 PAMAM dendrimer loaded with an EGFP plasmid, which showed much higher transfection efficiency compared to its nonfluorinated control, while also exhibiting very low cytotoxicity. Incredible transfection efficiency was obtained even at extremely low N/P ratio (ratio among nitrogen atoms‐ carrier‐ and P atoms – NA‐) and in medium containing 50% FBS, suggesting excellent serum resistance given by exposure of fluorinated moieties on the surface. Considering that fluorination modulates both the NA packing/unpacking ability of dendrimers and the interaction with biological media, the excellent transfection efficacy of this fluorinated dendrimer is to be attributed to the balance between several factors, such as fluoroalkyl chain length, number of grafted groups, residual charges, and degradability of the dendrimer [8].

To avoid accumulation issues related to the use of linear fluoroalkyl chains, short, branched fluorinated groups have been used to modify dendrimers. For example, fluorinated Janus‐type dendrimers (JD), whose structure consists of two domains of different polarity that can be tailored to control their self‐assembly, have been developed as ^19^F‐MRI‐traceable delivery carriers [72]. First, trifluoromethyl groups were added to JDs, showing promising results for ^19^F‐NMR/MRI‐traceable drug delivery vectors [73]. To further improve ^19^F‐MRI sensitivity and cargo‐loading ability, a branched fluorinated moiety bearing 27 F atoms (three perfluoro‐*tert*‐butyl groups) has been used as the hydrophobic portion of polyester dendrons of different generations, obtaining a self‐assembling system in aqueous media, whose behavior can be tuned by balancing fluorinated and hydrophilic domains [14, 72].

The molecular structure of the dendrimers strongly affects self‐assembly properties. In particular, first‐ and second‐generation fluorinated JD self‐assembled into nanoparticles stable over time, whereas the third‐generation analogue exhibited a time‐dependent behavior: freshly prepared formulations showed the presence of ~100 nm dendrimersomes, which progressively transformed into tubular structures after 48 h due to subtle interactions at the nano‐interface with cosolvent molecules. Moreover, ^19^F‐NMR signal of the third‐generation dendrimer was quenched after 48 h, suggesting limited mobility of the fluorinated groups in the tubular structures [72]. Considering the optimal balance between hydrophilic and hydrophobic parts, the 2^nd^ generation JD was then modified with amine groups to obtain an ionizable dendrimer (FJD_2_N), whose structure is shown in Fig. 4A. Cytotoxicity and transfection efficacy were investigated using pGLuc‐Basic 2‐FJD_2_N dendriplexes on multiple cell lines, as reported in Fig. 4B. Cell viability resulted dependent on cell type, showing lower cytotoxic levels than selected commercial benchmarks up to N/P = 40 (equal to a final [FJD_2_N] = 30 μm) for L929 and SH‐5YSY, while higher sensitivity to FJD_2_N was observed for the C2C12 cells with acceptable cytotoxicity up to N/P = 30 (equal to a final [FJD_2_N] = 23 μm). Overall, FJD_2_N can successfully transfect all tested cells with good viability up to 23 μm, corresponding to N/P ratio of 30. Moreover, both FJD_2_N and FJD_2_N‐siRNA dendriplexes exhibited ^19^F‐NMR signals, as shown in Fig. 4C, suggesting the possibility of using them as theranostic delivery vectors [19]. A dendritic amphiphile was also functionalized with this fluorinated moiety bearing three PFtB groups and its self‐assembly was investigated in comparison to its alkyl analogue [74]. While fluorinated amphiphiles formed hexosomes, the nonfluorinated ones self‐assembled into vesicles. Moreover, fluorination was proved to enhance drug loading ability, especially of fluorinated drugs, which disrupted the lattice ordering of the hexosomes to integrate themselves into the hydrophobic part of the nanostructure [74]. Although further optimization is needed for these systems, these results prove that dendrimer properties, such as structure and self‐assembly, can be finely tuned by adding branched fluorinated groups, leading to improved delivery efficacy. Furthermore, their ^19^F‐MRI signal allows them to be considered promising bioimaging tools [19, 74].

**Fig. 4:**
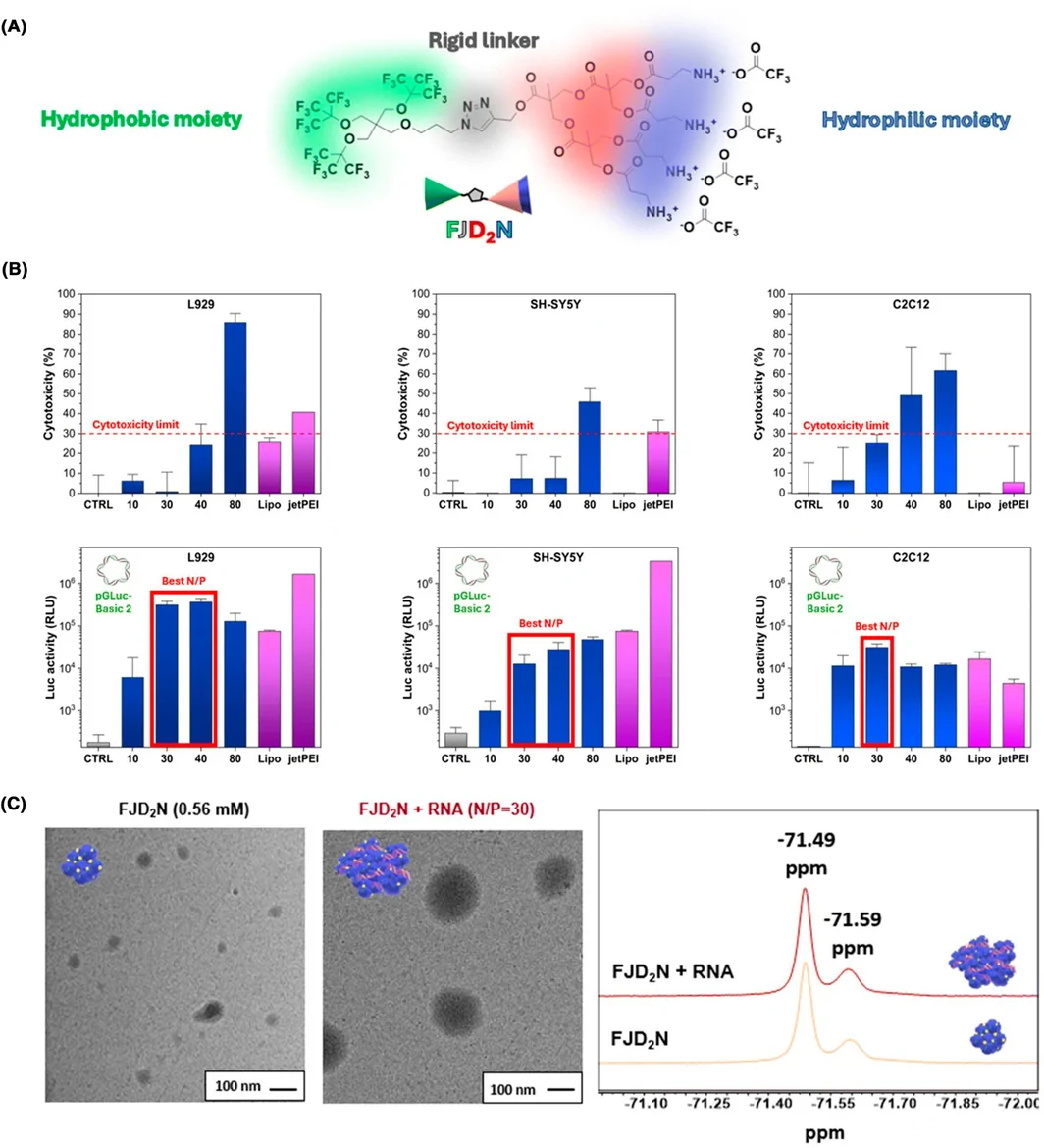
Structure, transfection efficacy and ^19^F‐MRI signal of FJD_2_N‐siRNA dendriplexes. (A) FJD_2_N chemical structure, highlighting the fluorinated hydrophobic part and the hydrophilic segment which allows NA binding; (B) Cytotoxicity (up) and transfection efficacy (down, measured in relative light units, RLU) on different cell lines using plasmid pGLuc‐Basic 2‐FJD_2_N dendriplexes at different N/P ratios. Cell lines: L929 (murine fibroblast from subcutaneous connective tissue), SH‐SY5Y (human neuroblastoma), and C2C12 (immortalized mouse myoblast). (C) Cryo‐TEM images (scale bar 100 nm) of 0.56 mm FJD_2_N and FJD_2_N‐siRNA dendriplexes (N/P = 30) in 150 mm NaCl and their ^19^F‐NMR signals: yellow –0.56 mm FJD_2_N; red ‐ FJD_2_N‐siRNA (Luciferase GL3) dendriplexes. Adapted from [19].

#### Lipids

In recent years, lipid‐based nanocarriers experienced significant growth both in research and industrial sectors, having gained approval as cancer treatments and COVID‐19 vaccines [75]. The reason behind their widespread usage can be found in their adaptability to encapsulate various therapeutic agents including small molecules, NAs, and antibodies [75]. However, lipid‐based carriers must overcome multiple intracellular and extracellular barriers to function *in vivo*, such as clearance, internalization in target cells, and cargo endosomal escape [76]. To improve delivery efficacy of these carriers, lipid tails have been functionalized with fluoroalkyl chains to obtain an enhanced molecular cone geometry, resulting in a facilitated transition to the preferred hexagonal II phase from a lamellar phase, which should promote a greater efficiency in disrupting endosomal membranes [77]. When considering cationic lipoplexes, serum‐free conditions are usually required to achieve high transfection efficacy [78]. Positive charges on the surface of cationic lipid/DNA complexes lead to nonspecific adsorption of negatively charged serum proteins [78], which compete for the surface of the positively charged lipids, resulting in DNA disassembly from the complex, and thus reduced transfection efficacy [79]. Lipoplexes with cationic lipids displaying a fluorous moiety, namely (CF_2_)_4_ or (CF_2_)_6_, close to the headgroup showed improved stability in biological media, and gene delivery occurred regardless of serum presence. However, these fluorinated carriers did not exhibit significant improvement of efficiency *in vitro* compared to the hydrogenated analogous [51]. Conversely, when functionalized with (CF_2_)_4_ and (CF_2_)_8_ groups similar lipoplexes showed higher *in vivo* efficiency compared to the nonfluorinated counterparts [50]. Fluoroalkyl chains, and (CF_2_)_8_ in particular, have also been introduced as termini in lipid tails, resulting in enhanced cytosolic delivery both *in vitro* and *in vivo* [12, 77]. In particular, a fluorinated ionizable lipid (F‐L319) was synthesized and used in LNP formulations in different mol% [12]. Hybrid formulations achieved higher EE%, cellular internalization, and transfection efficacy both *in vitro* and *in vivo* compared to the nonfluorinated control, while LNPs with only F‐L319 as ionizable lipid exhibited the lowest signal, probably due to the poor mRNA EE% [12]. These results clearly show how fluorination must be finely tuned to improve mRNA encapsulation efficiency, interaction with cell membranes, and transfection efficiency [12, 51].

In the last 2 years, branched fluorinated moieties have been introduced also into lipid nanoparticles. To shield the LNPs from interactions with proteins and other biomacromolecules, the presence of PEGylated lipids is now a standard in LNP formulations [51, 80]. To further improve the surface properties of LNP, a trifluoromethyl group was added to the terminal group of the PEG moiety of DSPE‐PEG lipid, rendering the surface of the LNP both hydrophobic and lipophobic, therefore limiting the interactions with serum proteins. Increasing the mol% of the fluorinated PEGylated lipid from 0% to 1.5% leads to higher eGFP expression in multiple cell lines, and further *in vivo* tests confirmed the enhanced transfection efficacy compared to their nonfluorinated counterpart, thus proving that shielding the surface of the LNP from interactions with proteins in biological media improves delivery efficacy [6]. Ionizable lipids have also been modified with fluorinated branched moieties: PFtB groups were added at the end of the lipid tails to improve transfection efficacy while also enabling real‐time tracking through ^19^F‐MRI. PFtB groups were added to lipids bearing 12 and 6 C atoms per tail, respectively, obtaining FC12 and FC6 (Fig. 5A) [13]. Results showed that LNPs containing 25 mol% FC12 significantly outperformed both nonfluorinated and 25 mol% FC6 LNPs in transfection efficacy. Molecular dynamics simulations showed that FC12 was able to enhance the conformational order of the lipid membrane, tightening lipid packing and reducing the average lipid area, while also increasing lateral mobility of the lipids. On the other hand, FC6 led to a more disordered lipid arrangement, thus facilitating water penetration and possibly compromising LNP integrity. Moreover, LNPs containing different amounts of FC12 were investigated and they all showed better transfection efficacy, cellular uptake, and endosomal escape than nonfluorinated LNPs, displaying correlation between FC12 percentage and transfection efficacy (Fig. 5B) [13]. However, FC12 exhibited a lower DNA‐binding ability compared to the nonfluorinated benchmark, therefore addition of high % of FC12 leads to poor EE%, hence identifying 25% FC12 LNP as the most practical and cost‐effective formulation and proving once again that fluorination needs to be finely tuned to achieve significant improvements [13]. Finally, since ionizable lipids are not exposed on the surface, but they are located inside the LNP, ^19^F‐MRI signal is strongly correlated to the disassembly of LNP and intracellular release of nucleic acids, thus providing a noninvasive method to monitor LNP localization and real‐time NAs release (Fig. 5C,D) [13].

**Fig. 5:**
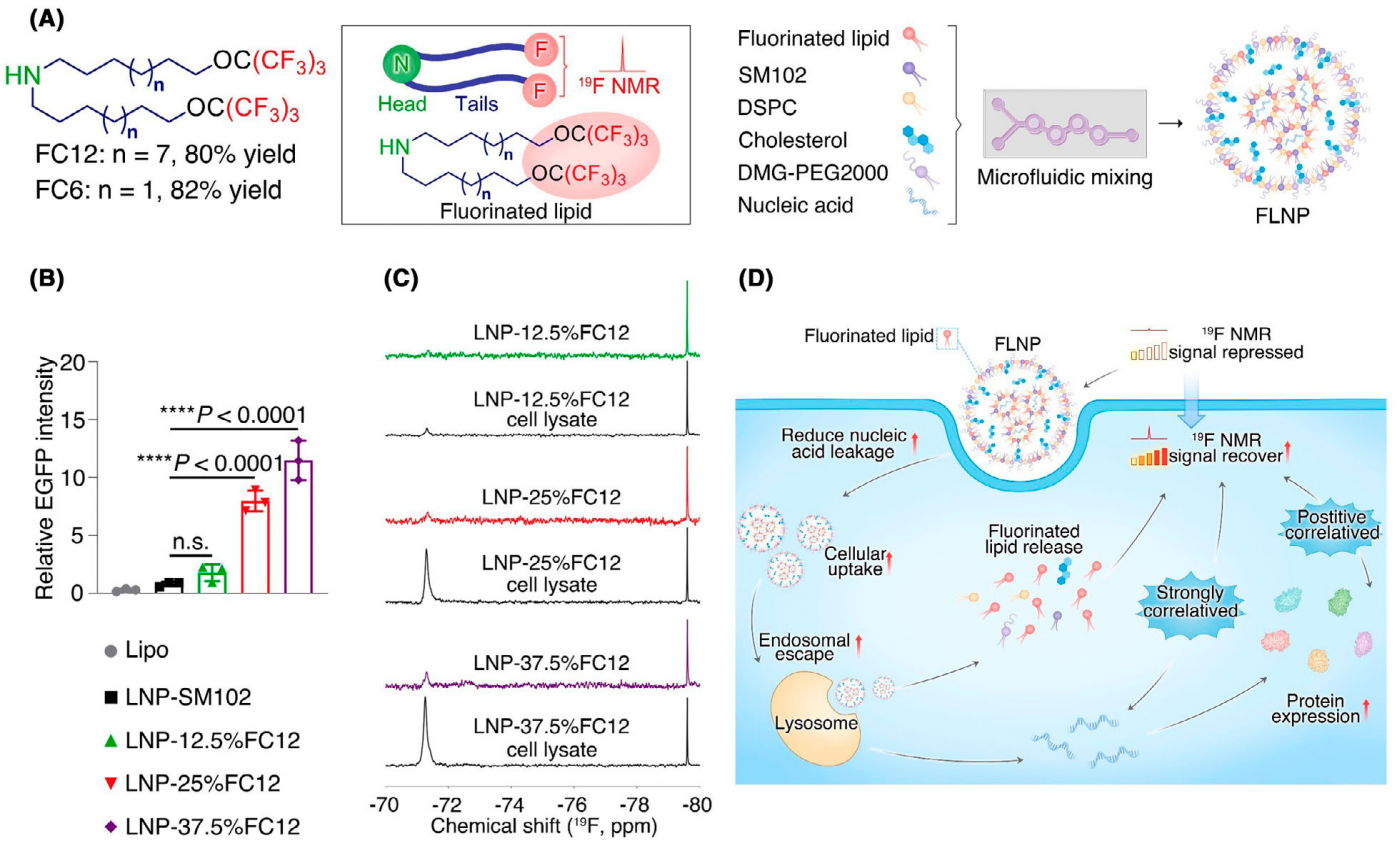
(A) Chemical structure of FC6 and FC12 and schematic representation of obtained LNPs; (B) *in vitro* transfection efficacy of lipid nanoparticles (LNPs) containing different mol% of FC12; (C) ^19^F‐NMR (nuclear magnetic resonance) signals of LNPs in cells before and after lysis; (D) schematic representation of the correlation between ^19^F‐NMR signal and protein expression. Adapted from [13].

## Conclusions and perspectives

In conclusion, fluorination has emerged as a powerful strategy to enhance the performance of drug delivery systems and biological therapeutics by improving key properties such as stability, membrane interactions, and delivery efficiency.

The exposure of fluorinated moieties to the biological environment reduces interaction with the proteins, simultaneously enhancing the affinity to lipid membrane for minimizing water–fluorine interactions. On the other side, fluorinated moieties within the lipid envelope will tend to segregate for ‘fluorous effect’ and more likely destabilize the lipid membrane in the endosomes.

However, the design of fluorinated systems requires careful optimization to achieve the appropriate balance between hydrophilicity, lipophilicity, and the degree of fluorination, thereby maximizing biological performance while avoiding unnecessary structural complexity.

While the environmental sustainability of fluorinated compounds is increasingly considered, it is important to distinguish fluorinated pharmaceuticals and adjuvants from the industrial PFAS associated with widespread environmental persistence and bioaccumulation.

At the same time, the sustainability of fluorinated compounds is receiving increasing attention in light of concerns surrounding the persistence, bioaccumulation, and potential toxicity of certain PFAS. Although fluorinated pharmaceuticals and adjuvants contribute only marginally to the overall fluorinated chemical burden, minimizing fluorine content where possible or employing more sustainable fluorinated motifs, such as branched fluorinated chains, may further improve the sustainability of therapeutic platforms, while also providing valuable imaging capabilities, including ^19^F‐MRI and Raman imaging.

The main message of this minireview is that, although fluorination has consistently been shown to improve the performance of therapeutic agents, the molecular mechanisms underlying the favorable interactions of fluorinated moieties with biological systems—including proteins, cellular membranes, and endosomal membranes—remain only partially understood. A deeper mechanistic understanding of these interactions is therefore essential for the rational design of next‐generation fluorinated therapeutics and drug delivery platforms.

Addressing this knowledge gap will require the integration of advanced physicochemical characterization techniques capable of probing bio–nano interactions with high spatial and structural resolution. Neutron and X‐ray scattering techniques offer unique opportunities to investigate interactions with proteins and other biomacromolecules, while neutron and X‐ray reflectometry, combined with well‐defined model membrane systems, can provide detailed insights into the interfacial processes governing the interaction of fluorinated nanomaterials with biological membranes. Such mechanistic studies will ultimately enable the development of more effective, safer, and environmentally responsible fluorination strategies for biomedical applications.

## Conflict of interest

The authors declare no conflict of interest.

## Author contributions

MB, BLB, and FBB conceived the contents of the manuscript. MB and FBB wrote the first draft of the manuscript. BLB revised the manuscript and prepared the figures with MB.

## Data Availability

Data sharing is not applicable to this article as no new data were created or analyzed in this study.
